# First Immature of the New World Treehopper tribe Thuridini (Hemiptera, Membracidae, Smiliinae) with a new synonym, a new combination, and a new country record

**DOI:** 10.3897/zookeys.557.6602

**Published:** 2016-01-28

**Authors:** Stuart H. McKamey, Mitchell J. Porter

**Affiliations:** 1 USDA/ARS Systematic Entomology Lab, c/o NMNH MRC-168, Smithsonian Institution, Washington, DC 20560, USA; 2 Department of Biology, University of Maryland, College Park, MD 20742

**Keywords:** Thuris, Parantonae, nymph, syn. n., comb. n., Ecuador, Venezuela

## Abstract

The species *Thuris depressus* Sakakibara (1975) is proposed as a **syn. n.** of *Thuris binodosus* (Goding 1926), **comb. n.** The distribution of the genus is expanded from Brazil and Peru to include Ecuador and Venezuela, and the immature is described based on 75 characters.

## Introduction

Adult treehoppers (Membracidae, Aetalionidae, and Melizoderidae) are well known for the expanded, often extravagantly developed pronotum common to nearly all of the more than 400 genera and 3,000 species (McKamey 1998). The immature stages are poorly known, but in addition to the nascent enlarged pronotum, nymphs are usually covered with various arrangements of large spinelike structures (scoli) or smaller setae with tuberculate or stalked bases (chalazae) on the head, all thoracic segments, and the abdomen (Fig. 12). These structures are usually absent in the adults. Indeed, treehopper immatures show a vast array of structures that to a large extent have evolved independently of the adult forms. The nymph of *Thuris* Funkhouser (1943), a genus containing two valid species, has never been described. The description below is based on 75 nymphal characters and 322 possible character states exhibited among members of the subfamily Smiliinae, which includes the monobasic tribe Thuridini. Because many of the characters are conditions of the scoli and enlarged chalazae (direction, size, which segments, etc.), the description below seems abbreviated but covers all characters.

## Materials and methods

Late instars of Membracidae are usually sturdy enough to maintain their form when dried, so a pinned specimen was used to determine characters. The only character that would likely be affected is the length of abdominal segment IX relative to other body parts, due to contraction upon drying.

Because some form of parental care or at least aggregation of nymphs is widespread among treehoppers, for many subfamilies it is easy to associate adults, nymphs, and egg masses. In the case of solitary taxa, repeated adult-nymph-host association, rearing, and in a few cases the extrapolation of the miniature pronotom have been used to associate adults and nymphs. In the case of *Thuris*, an aggregation of four late instars and four adults was collected, suggesting that the genus is subsocial.

The voucher (INHS) has the label “Immatures Project Voucher, McKamey et al. 2015” and the species name.

Images were captured with a Microvision system and Cartograph 8.0.6 automontage software and adjusted in Adobe Photoshop.

The terminology, 75 characters and 322 potential character states are the same used for Amastrini (McKamey et al. 2015). The character descriptions and data are posted online on the USDA/ARS Systematic Entomology Laboratory website (McKamey 2015).

**Specimen repositories are as follows**

DZUP
 Brazil, Paraná, Curitiba, Universidade Federal do Paraná, Museo de Entomologia Pe. Jesus Santiago Moure 


INHS
USA, Illinois, Campaign, Illinois Natural History Survey 


NCSU
USA, North Carolina, Raleigh, North Carolina State University 


USNM
USA, District of Colombia, Washington DC, National Museum of Natural History 


## Results

### Thuris binodosus

Taxon classificationAnimaliaHemipteraMembracidae

(Goding) comb. n.

Parantonae binodosa Goding 1926: 108 (Figs 1–4, 8; holotype USNM)
Thuris depressus Sakakibara 1975: 843, **syn. n.** (Figs 5–7; holotype DZUP)

#### Notes and new distribution.

**Figures 1–7.**
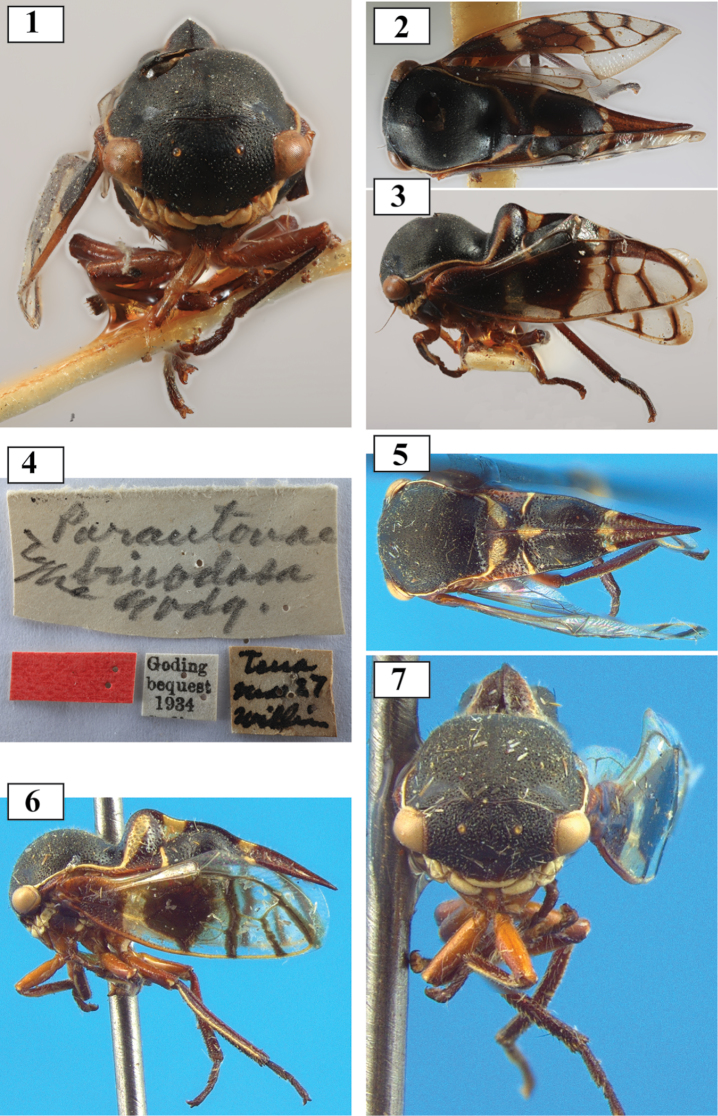
*Thuris binodosus* (Goding), comb. n. **1–4** *Parantonae binodosa* holotype and labels **5–7** *Thuris depressus*, syn. n., holotype.

**Figure 8.**
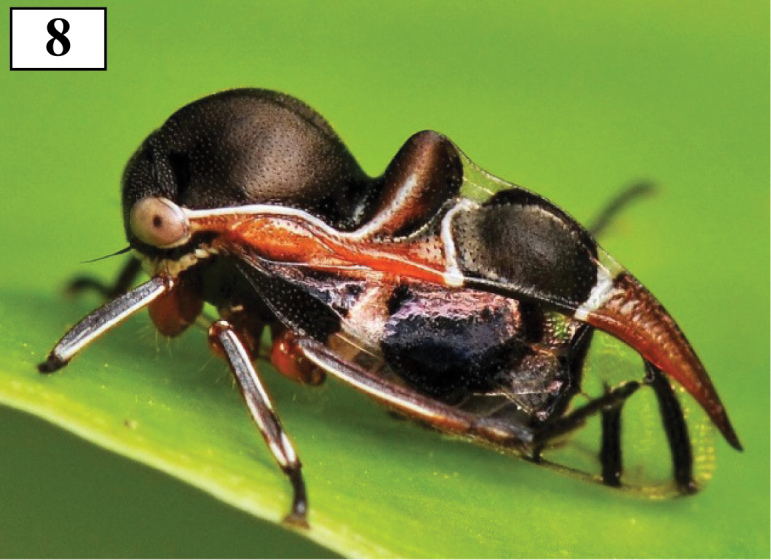
*Thuris binodosus*, live shot, Ecuador. Photo Milan Kozánek, with permission (http://www.kozanek.com/en/insects/18/?slide=559).

Examination of the holotype of *Parantonae binodosa* Goding (1926), from Tena, Ecuador (Napo Province) (Figs 1–4) revealed a match with the holotype of *Thuris depressus* Sakakibara (1975) (Figs 5–7), resulting in the classification above. An aggregation of the species has also been observed in Ecuador in Coca (= Puerto Francisco de Orellana, Napo Province) and a solitary adult was found in (Fig. 8) Otoyaku, Santa Clara (Pastaza Province). The Venezuelan *Thuris* specimen (of *Thuris fenestratus* Funkhouser) was collected in Amazonas state.

#### Description of fifth instar

(Figs 9–11).

**Figures 9–12.**
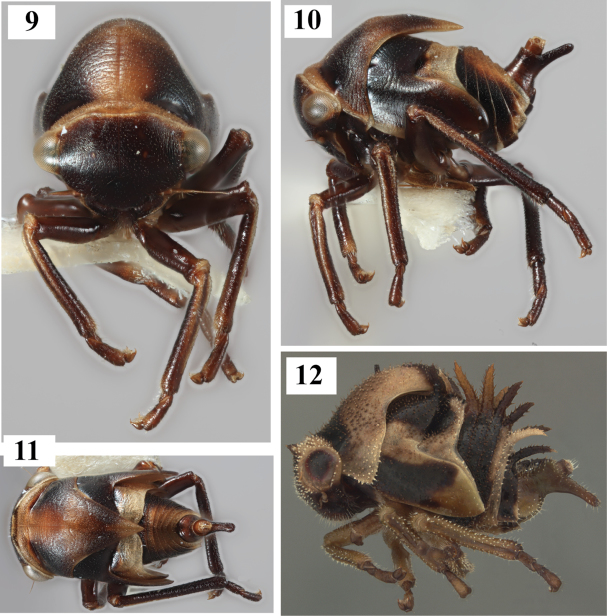
**9–11** Fifth instar of *Thuris binodosus* (Goding) **12** Fifth instar of *Neotynelia* sp., which also exhibits a ventrally extended abdominal segment IX.

#### Diagnosis.

Body densely covered with short setae, but lacking chalazae and scoli; apex of abdominal segment IX narrowly extended ventrally far beyond dorsal extension.

#### Description.

***Overall body*.** Length 2.50 mm, maximum width 1.25 mm. Cross-section of abdomen subcircular; chalazae on thorax and abdomen absent; waxlike substance absent; dorsal contour of abdomen in lateral view curvilinear (Fig. 10); overall body in dorsal view elongate (distinctly longer than wide). Coloration a combination of black and brown, most pale on premetopidium, metanotum, margins of tibia, and mid dorsally from pronotum to abdominal tergum VIII. ***Head*.** Scoli, dorsal or anterior rounded protuberances, and chalazae absent; compound eye surface setae present; frontoclypeus not extending over central margin of eye. ***Prothorax*.** Premetopidium and postmetopidium scoli absent; metopidial sulcus deeply incised; posterior extension of pronotum acute, surpassing posterior margin of metanotum but not attaining abdominal apex; dorsal pronotal single medial, suprahumeral, and humeral horn buds absent; pronotal lateral margin straight. ***Mesothorax*.** Dorsal projections absent; forewing pad anterior costal margin form straight, surface and costal chalazae absent. ***Metathorax*.** Dorsal projections absent. ***Legs*.** Tibia without chalazae; prothoracic tibia form subcylindrical, without defined margins; metathoracic tarsal length longer than pro- and mesothoracic tarsal length; pro- and mesothoracic first tarsomeres distinctly shorter than their second tarsomeres (Fig. 9); metathoracic first tarsomere subequal in length to its second tarsomere (Fig. 10). ***Abdomen*.** Terga III-VIII ventrolateral margins without enlarged chalazae or other lateral extensions, lateral longitudinal rows of enlarged chalazae or scoli between mid dorsal line and ventrolateral margins not manifested. ***Segment IX*.** Distal half tubular in cross-section; dorsal length subequal to length of segment V-VIII; dorsal projections before and at apex absent; ventral extension narrow and distinctly longer than dorsal extension (Fig. 10); fused portion of segment IX distal to unfused portion; unfused portion distally not bifurcate.

#### Discussion.

The nymph of *Thuris binodosus* is exceptionally devoid of the chalazae and scoli that adorn most treehopper nymphs, including those of the related tribe Tragopini, and that is in itself distinctive. Another diagnostic feature is the ventrally extended abdominal tergum IX, which among other membracid immatures only occurs in a few taxa. Although the ventral extension is distinctly longer than the dorsal extension in *Todea cimicoides* (Coquebert) and *Colisicostata albata* (Tode) of Tragopini, *Phormophora maura* (Fabricius) of Polyglyptini, and most Aetalionidae, only in *Neotynelia* Creão-Duarte & Sakakibara (2000; Amastrini) is the extension digitiform as in *Thuris binodosus*. This digitiform ventral extension feature was not used in the phylogenetic study by Dietrich et al. (2001) and, perhaps because of this, *Thuris* did not form a clade with Amastrini. *Neotynelia* differs in always bearing large scoli on the abdomen at least (Fig. 12). Deitz (1975) established Thuridini as a tribe based in large part on the absence of cucullate setae in metathoracic tibial row I. Unfortunately, these basally covered setae so ubiquitous among membracid adults are absent in the nymphs. The subequal lengths of the metathoracic first and second tarsomeres, due to an extraordinarily long first tarsomere, is unusual but shared with other taxa such as Tragopini (McKamey et al., in prep) and is reflected in the similarly long first tarsomere (of three total) in *Thuris* adults.

#### Specimens examined.

*Parantonae binodosa* holotype (USNM). *Thuris fenestratus* Funkhouser holotype (USNM). Additional material of *Thuris binodosus*: 1 nymph. ECUADOR: Napo, Coca 24-VIII-1988, C. H. Dietrich #86, INHS Insect Collection 776, 690 (INHS). Not examined but same collection lot as examined nymph: 1 nymph, 1 adult (INHS), 2 nymphs, 3 adults (NSCU). Specimens of *Thuris fenestratus*: 1 adult. VENEZUELA: T. F. Amazonas, San Carlos de Rio Negro, 24-I-1985, P. & P. Spangler, R. Faitoute, W. Steiner (USNM).

## Supplementary Material

XML Treatment for Thuris binodosus
